# Hepatitis B-Associated Symptomatic Iron Overload, with Complete Resolution after Nucleoside Analogue Treatment

**DOI:** 10.1155/2021/8407257

**Published:** 2021-12-27

**Authors:** Tze Tong Tey, Richard Yiu, Wei Qiang Leow

**Affiliations:** ^1^Department of Gastroenterology & Hepatology, Sengkang General Hospital, Singapore; ^2^Department of Haematology, Sengkang General Hospital, Singapore; ^3^Department of Pathology, Singapore General Hospital, Singapore

## Abstract

Symptomatic iron overload and hyperferritinemia are rarely mentioned as complications of chronic hepatitis B infection. We report a case of a 70-year-old woman who presented with symptoms of iron overload including aches in the calves, fatigue, poor appetite, and low mood. Laboratory results showed a serum ferritin of 2449 *μ*g/L and transferrin saturation of 74%. Her symptoms completely resolved with hepatitis B antiviral treatment. Serum ferritin and transferrin saturation also normalized. Symptomatic iron overload is a rare yet clinically important complication that can result from chronic hepatitis B infection.

## 1. Introduction

Hepatitis B is a common infection worldwide which leads to significant morbidity and mortality through liver cirrhosis and its complications [1]. One of the lesser known associations of hepatitis B infection is iron overload [2]. We report an unusual case of a patient who presented with severe symptomatic iron overload due to hepatitis B which fully resolved after hepatitis B treatment.

## 2. Case Presentation

A 70-year-old woman with no known past medical history presented with bilateral calf aches for three months which were so severe that she developed insomnia and a low mood. She also had associated symptoms of fatigue and loss of appetite without weight loss.

Her blood tests on admission revealed elevated aspartate transaminase (AST) 547 [12–42 U/L], alanine transaminase (ALT) 485 [6–66 U/L], bilirubin 41 [7–32 umol/L], and albumin 34 [40–51 g/L]. There was a markedly elevated serum ferritin of 2449 [13–150 *μ*g/L] and transferrin saturation of 74% [<50%]. The rest of her iron studies showed transferrin 1.3[2–3.6 g/L] and serum iron 25 [8–32 *μ*mol/L]. Hemoglobin was 10.1 [12–16 g/dL], and the mean corpuscular volume (MCV) was 96.3 [78–98 fL]. White blood cell count was 5.37 [4–10 × 10^9^], and platelet count was 82 [140–440 × 10^9^/L]. Serum creatinine was 51 [44–80 *μ*mol/L], plasma glucose was 4.7 [3.9–11 mmol/L], and prothrombin time was 13.2 [9.9–11.4 sec].

She did not drink alcohol and did not take iron supplements and herbal or traditional medication. She did not have previous blood transfusions and had no family history of iron overload disorders. The thyroid function test was normal.

Physical examination revealed no stigmata of chronic liver disease. Abdominal exam showed no hepatosplenomegaly or ascites.

Her serology test results were as follows: hepatitis B surface antigen positive and hepatitis C IgG negative. Hepatitis B DNA load was 6.83 log IU/ml, hepatitis B e antigen negative, and e antibody positive. Abdominal ultrasound showed a liver with coarsened echotexture and irregular nodular outline, with no suspicious focal lesion. Spleen size was normal.

Echocardiography was unremarkable, showing normal left ventricular systolic and diastolic function and no features of cardiac hemochromatosis.

Liver biopsy showed cirrhosis, with several small hepatocytic nodules rimmed by thick and inflamed Masson trichrome-positive fibrous septa. No significant cholestasis or steatosis was present. Perl's stain showed iron deposits within Kupffer cells, with no significant iron deposition within the hepatocytes, consistent with secondary causes of iron overload and not primary hemochromatosis. A diagnosis of immune-active chronic hepatitis B was made, and the patient was prescribed entecavir 0.5 mg once a day.

She was reviewed in the clinic 3 months later. The iron overload symptoms including severe calf aches, insomnia, and low mood had resolved completely. Ferritin decreased to 559 [13–150 *μ*g/L], and transferrin saturation decreased to 48% [<50%]. At her subsequent visit 9 months after the initial diagnosis, ferritin decreased to 295 [13–150 *μ*g/L], and transferrin saturation decreased to 35% [<50%]. Her hepatitis B DNA load decreased to <10 IU/ml (<1 Log IU/ml). Liver transaminases decreased to AST 59 [12–42 U/L] and ALT 41 [6–66 U/L]. Bilirubin was 13 [7–32 umol/L], and albumin was 43 (40–51 g/L).

## 3. Discussion

The liver plays an important role in iron metabolism. It is the major production site of the iron regulatory peptides: ferritin, transferrin, and hepcidin. Liver derangements, therefore, have a direct effect on iron regulation [3, 4]. The exact physiological mechanism of how iron overload occurs is still an area of ongoing research. One theory states that hepatocytes which have been damaged by viral hepatitis undergo necrosis, and the iron released is subsequently scavenged by Kupffer cells [5, 6].

The patient described in this case had extremely elevated levels of ferritin and transferrin saturation, remarkably higher than what other studies have reported in chronic hepatitis B. A study by Gao et al. found that the mean ferritin was 550 *μ*g/L, and mean transferrin saturation was 45% in chronic hepatitis B patients compared to 130 *μ*g/L and 31% in healthy controls, respectively [2].

When evaluating a patient with a ferritin level this high, it is reasonable to consider hereditary hemochromatosis as a differential diagnosis. However, taking into account the Asian ethnicity of this patient, hereditary hemochromatosis was considered unlikely as the HFE gene mutation is known to be rare in the Asian population [7, 8]. A large population-wide study of 99,711 participants by Adams et al. on HFE genotypes showed that the prevalence of the C282Y homozygous mutation (hereditary hemochromatosis) was 0.44% in whites, 0.027% in Hispanics, and 0.000039% in Asians [9]. Furthermore, the liver biopsy result showing iron deposits in the Kuppfer cells but not in hepatocytes also supported the diagnosis of secondary iron overload [10].

After a detailed medical and drug history was taken to exclude secondary causes of iron overload, the most likely cause was determined to be chronic hepatitis B with active inflammation, and antiviral treatment with entecavir was started. There was a significant improvement in the iron indices after 3 months of treatment, and complete normalization was seen at 9 months. Her iron overload symptoms also resolved completely, and her mood was back to normal.

This case illustrates that symptomatic iron overload is a rare yet clinically important complication of chronic hepatitis B. When managing such patients, treatment should be directed at hepatitis B instead of instituting phlebotomy or iron chelation therapy, which this case showed were not needed and they also have multiple side effects [5]. Lastly, in the management of chronic hepatitis B, physicians should be mindful of and watch out for iron overload symptoms which are insidious and easily missed [11].

## Data Availability

Data regarding this case/manuscript are available upon request from the corresponding author.
